# Frontal midline theta transcranial alternating current stimulation enhances early consolidation of episodic memory

**DOI:** 10.1038/s41539-024-00222-0

**Published:** 2024-02-16

**Authors:** Limor Shtoots, Asher Nadler, Roni Partouche, Dorin Sharir, Aryeh Rothstein, Liran Shati, Daniel A. Levy

**Affiliations:** 1 Baruch Ivcher School of Psychology, Reichman University, Herzliya, 4610101 Israel; 2 Department of Psychology, Palo Alto University, Palo Alto, CA 94304 USA

**Keywords:** Long-term memory, Consolidation

## Abstract

Evidence implicating theta rhythms in declarative memory encoding and retrieval, together with the notion that both retrieval and consolidation involve memory reinstatement or replay, suggests that post-learning theta rhythm modulation can promote early consolidation of newly formed memories. Building on earlier work employing theta neurofeedback, we examined whether theta-frequency transcranial alternating stimulation (tACS) can engender effective consolidation of newly formed episodic memories, compared with beta frequency stimulation or sham control conditions. We compared midline frontal and posterior parietal theta stimulation montages and examined whether benefits to memory of theta upregulation are attributable to consolidation rather than to retrieval processes by using a washout period to eliminate tACS after-effects between stimulation and memory assessment. Four groups of participants viewed object pictures followed by a free recall test during three study-test cycles. They then engaged in tACS (frontal theta montage/parietal theta montage/frontal beta montage/sham) for a period of 20 min, followed by a 2-h break. Free recall assessments were conducted after the break, 24 h later, and 7 days later. Frontal midline theta-tACS induced significant off-line retrieval gains at all assessment time points relative to all other conditions. This indicates that theta upregulation provides optimal conditions for the consolidation of episodic memory, independent of mental-state strategies.

## Introduction

Recent years have seen an increasing recognition of the key role of brain oscillations in memory processes^1^. Higher theta EEG power during both encoding and retrieval is associated with better memory performance^2–5^. Additionally, widespread cortical and cortico-hippocampal theta phase synchronization is found to characterize effective encoding and has been proposed to facilitate simultaneous activation of neural assemblies^6^. This evidence, together with the notion that both retrieval and consolidation involve memory reinstatement or replay, suggests that post-learning theta rhythm modulation might promote early consolidation of newly formed memories. Support for this contention comes from a report that disruption of theta oscillations during post-learning REM sleep in rodents abolished consolidation of hippocampus-dependent contextual fear conditioning and novel object place recognition^7^. Relatedly, it was reported that event-related changes in spectral theta power during pre-sleep memory formation in humans were associated with greater overnight improvement in subsequent cued recall of word pairs^8^. It remains to be determined whether theta upregulation during post-learning waking would benefit subsequent memory.

In our previous research^9,10^, we used EEG neurofeedback (NFB) to enable participants to selectively increase spectral theta power following episodic memory encoding, while other participants engaged in low-beta-focused NFB or passively viewed a neutral nature movie, as active and passive control groups, respectively. Free recall assessments following the interventions, 24 h later, and 7 days later all indicated that theta upregulation via NFB benefited episodic memory consolidation. The degree of benefit to memory was correlated with the extent of theta power modulation but not with other spectral changes.

A mechanistic question raised by these findings relates to the possibility that theta upregulation via NFB improved memory because mental strategies employed to upregulate theta power are more conducive to avoiding post-training interference than those yielding greater beta power. Lower resting theta/beta ratios have been found to be related to better attentional control^11^, and increased alertness, manifested by faster responses to target visual stimuli, is accompanied by higher EEG activation in the beta band^12^. In contrast, theta activity has been associated with meditative states and drowsiness^13,14^. NFB groups might have used different mental-state strategies to increase their band-power targets (relaxation for theta vs. concentration for beta). Accordingly, the advantage of the theta condition might have resulted from its indirectly enabling neural plasticity processes to achieve completion by preventing retroactive interference and minimizing competition for brain resources. It, therefore, remains to be determined whether theta power upregulation in itself is sufficient to engender more effective consolidation of newly formed memories.

One method that enables theta rhythm activity upregulation without those confounds is transcranial alternating current stimulation (tACS), in which an external sinusoidal oscillating current applied to the scalp temporally entrains endogenous neuronal firing within the stimulation frequency possibly inducing long-term synaptic changes through spike-time-dependent plasticity^15,16^. Since previous studies indicated beneficial effects of tACS during encoding or retrieval on memory performance^17,18^ (but see^19,20^), we explored whether it would have parallel effects when applied during the initial post-learning consolidation window. Importantly, as opposed to NFB which requires strategic control by participants, tACS induces neural entrainment that is not linked with particular cognitive/affective strategies. Finding differential theta entrainment advantage for subsequent memory would indicate the specificity of theta rhythm benefit to episodic memory consolidation.

Since tACS (as opposed to NFB) is focused on particular scalp locations, the selection of the most effective montage for the stimulation of brain areas involved in consolidation processes is important. Previous work indicated that memory-related cortical-hippocampal networks can be enhanced noninvasively based on interactions between the hippocampus and angular gyrus during encoding^21^. Since consolidation via memory reinstatement or replay seemingly requires reactivation of cortico-hippocampal interactions initially engendered during encoding, we used a parietal montage for one experimental group.

Other previous research indicated the importance of frontal midline theta for mnemonic processes^22^. While it is difficult to associate scalp-recorded frontal midline theta power with specific cortico-hippocampal interactions, there is evidence indicating that such interactions occur in mnemonic contexts, and indeed it is proposed that theta oscillations organize communication and information transfer between MTL and PFC regions^23^. For example, intracranial recording data indicated that a task-modulated increase in theta coherence values between PFC and MTL was seen during free recall^24^, and ∼3-Hz theta oscillations that support MTL-PFC amplitude coupling immediately preceding memorandum onset were related to age-associated differences in recognition performance^25^. Furthermore, our previous work on the effects of theta NFB on the consolidation of episodic and spatial memory also indicated the importance of frontal midline theta in participants who had improved memory performance^9,10,26^. Therefore, we chose to use a frontal midline montage for another experimental group. As in our prior studies of theta effects on consolidation using neurofeedback^9,10,26^, a frontal beta condition served as an active control condition, in which participants underwent stimulation at the same amplitude but at a different frequency. A sham stimulation condition served as a passive control condition.

A second goal of the current research was to examine the specificity of theta upregulation benefits to consolidation as opposed to retrieval processes. In our prior work with NFB^9,10,26^, the initial test to assess the effects of theta or beta band upregulation on memory was conducted immediately after the NFB intervention. That raises the question of whether the theta upregulation benefitted consolidation of the prior learning or, alternatively, had some salutary effect on the initial retrieval. While we found that theta NFB benefitted not only the initial test but also tests at delays of a day and a week, those subsequent benefits might have resulted from the initially better retrieval. Therefore, in the current study, we postponed the initial test to avoid stimulation aftereffects on retrieval. While several studies have reported persistent changes in resting alpha oscillatory power following tACS at that frequency (e.g.,^27–29^), reports of persistence in theta increase are rarer^30,31^. Nevertheless, we took steps to avoid such possible aftereffects, guided by the finding that even the more common alpha band effects were reported to have washed out once 70 min had passed after tACS^32^. For added caution, in the current study, all groups were initially tested only 120 min after the intervention, by which time theta stimulation aftereffects would arguably have washed out. Thus, any benefits to the memory of theta upregulation would not be attributable to benefits to retrieval processes but rather to the consolidation of learning.

If either or both loci (posterior parietal or midline frontal) of theta-focused stimulation during a post-learning time window of consolidation prove to benefit subsequent memory relative to a sham condition or to an active control condition (the Beta Frontal group), this would indicate that the beneficial effects of theta upregulation, as was found for NFB^9,10,26^, cannot be attributed to strategic factors such relaxation or manipulation of attentional focus. Rather, it would indicate that theta oscillatory activity is inherently beneficial to consolidation processes. As detailed below, we found that to be the case.

## Results

Four randomly assigned groups of participants viewed object pictures followed by a free recall test during three study-test cycles. They then engaged in tACS (frontal theta montage/parietal theta montage/frontal beta montage / sham) for a period of 20 min, followed by a 2-h break (a washout period to eliminate tACS after-effects between stimulation and memory assessment). Free recall assessments of benefits to subsequent memory attributable to the enhancement of consolidation processes were conducted after the break, 24 h later, and 7 days later.

### tACS effects on free recall

**Fig. 1:**
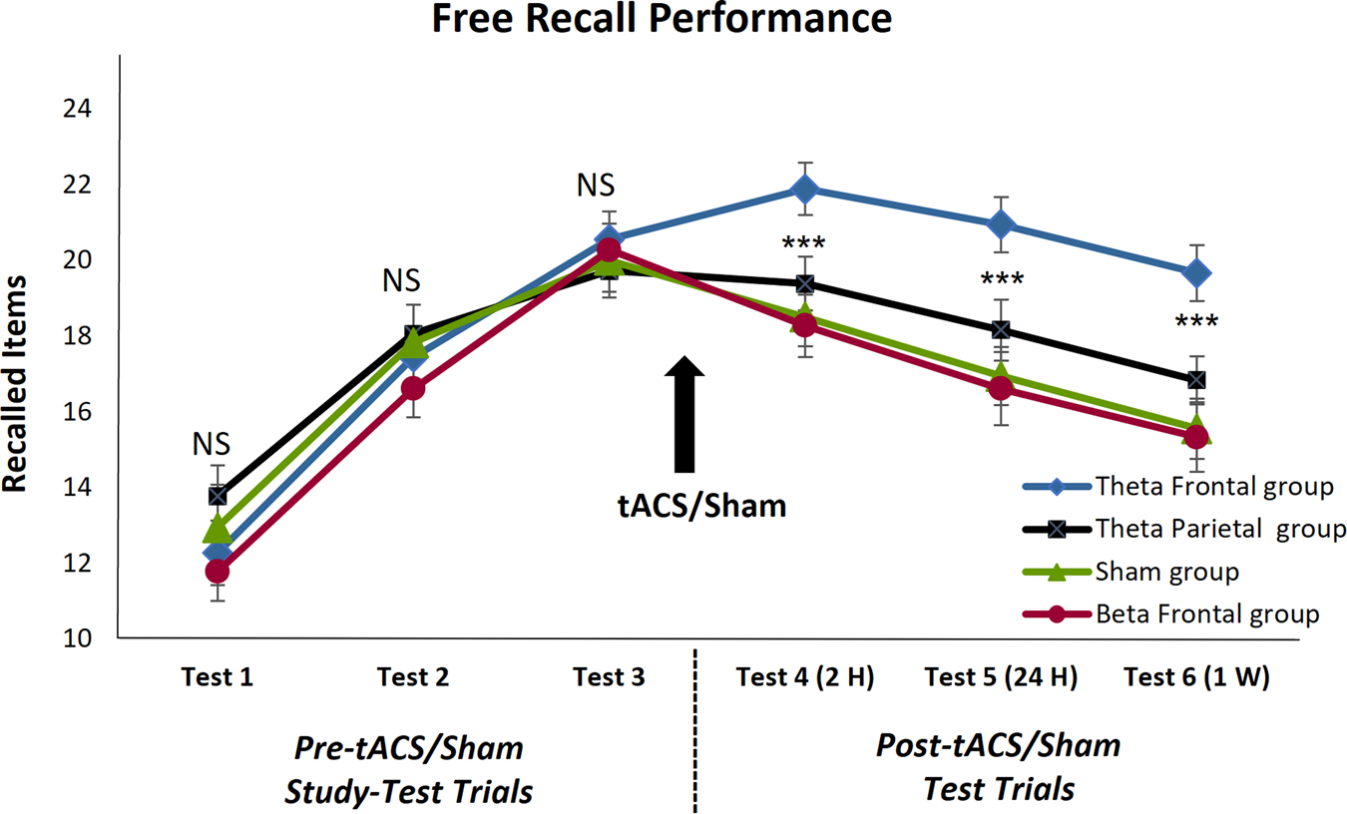
Free recall scores during each stage of the experiment for the Theta Frontal, Theta Parietal, Beta Frontal, and Sham groups. Error bars indicate Mean ± SEM; NS non-significant; ***, *p* = 0.001 (repeated measures ANOVA), for the main effect difference between Theta Frontal and all other groups. For effect sizes of the differences between Theta Frontal and the other groups in each test, see Table 1.

tACS/Sham effects on free recall performance for the four experimental groups are presented in Fig. 1. We first confirmed, using Kolmogorov–Smirnov and Shapiro–Wilk tests, that performance scores in all of the three baseline tests, as well as in all critical post-intervention tests, were normally distributed. We then conducted repeated measures ANOVA on the number of correctly recalled items, with within-subjects repeated factor of test number and between-subjects factor of group (Frontal Theta, Parietal Theta, Frontal Beta, Sham), to examine whether there were baseline differences in the initial three, pre-tACS/Sham study-test trials. Maunchly’s Test of Sphericity indicated that corrected degrees of freedom were required; the Greenhouse-Geisser correction was applied, as seen in the following parameters. The repeated measures ANOVA revealed a main effect of test number, *F*(1.626,110.543) = 313.81, *p* < 0.001, partial *η*^2^ = 0.822, and no main effect of group, *F*(3,68) < 1.0. However, there was a marginal group × test interaction, *F*(4.877,110.543) = 2.165, *p* = 0.065. We, therefore, followed up with a one-way ANOVA examining group free recall scores for the third and final pre-intervention test; this revealed no group differences, *F* < 1.0. This indicates that later effects of tACS cannot be attributed to prior group differences in baseline memory ability or initial learning.

We then examined the impact of tACS/Sham intervention on subsequent free recall trials. Repeated measures ANOVA for free recall performance on tests 4–6 indicated a main effect of the test, *F*(2136) = 65.00, *p* < 0.001, partial *η*^2^ = 0.489 (reflecting natural forgetting over time, as anticipated), and a main effect of group *F*(3,68) = 6.475 *p* = 0.001, partial *η*^2^ = 0.222. There was no interaction between the test and group *F*(6136) < 1.0. We further examined the relationship between the performance of the groups at each post-tACS/Sham assessment point (exams 4, 5, and 6) by calculating the effect size for the Theta Frontal group compared to the three remaining experimental groups, using Hedges’ *g*. Our results reveal a strong effect size (all values are above 0.8), while comparisons between other conditions revealed much smaller effect sizes (Table 1).

**Table 1:** Effect sizes in Hedges’ *g* values

Group Differences	Test 4 (2 H)	Test 5 (24 H)	Test 6 (1 W)
Theta Frontal vs. Theta Parietal	0.85	0.83	0.98
Theta Frontal vs. Beta Frontal	1.12	1.19	1.23
Theta Frontal vs. Sham	1.11	1.27	1.26
Theta Parietal vs. Beta Frontal	0.34	0.42	0.45
Theta Parietal vs. Sham	0.29	0.39	0.40
Beta Frontal vs. Sham	0.06	0.08	0.08

Another feature of the results presented in Fig. 1 is that only the Theta frontal group displayed a significant off-line gain in performance, as confirmed by a paired sample *t*-test, *t*(17) = 6.234, *p* < .001.

### Reported sensations related to transcranial electrical stimulation

Participants in all four groups did not differ in their level of itching, as revealed by Univariate GLM analysis, *F*(3,68) = 1.507, *p* = 0.221; pain, *F*(3,68) < 1.0; burning sensation, *F*(3,68) = 1.575, *p* = 0.203; warmth/heat, *F*(3,68) < 1.0; fatigue/decreased alertness, *F*(3,68) < 1.0; and general feeling, *F*(3,68) < 1.0, during the 20 min of tACS/Sham. However, there was a significant difference in the reports of the level of metallic/iron taste *F*(3,68) = 4.96, *p* = 0.004. Sham group participants reported significantly lower levels of metallic/iron taste in comparison to the Parietal Theta tACS group, *p* = 0.006; which aligns with the fact that metallic/iron taste is a known side effect of transcranial electrical stimulation^33^; however, the differences between the Sham and other groups were not significant.

### Participants were blind to group assignment

After each experiment was completed, we asked participants if they thought that they were part of the control group or part of the experimental group. 78% of the participants in the Theta Frontal group, 72% of the participants in the Theta Parietal group, 67% of the participants in the Beta Frontal group, and 83% of the participants in the Sham group thought that they were part of the experimental group. Our results indicate, via a Chi-square test, that participants in all four groups did not differ in their answers, *χ*^2^(3,72) = 1.48, *p* = 0.69, suggesting that they were blind to the condition to which they were assigned.

## Discussion

The current study demonstrates enhancement of episodic memory, assessed as free recall performance, following the application of frontal midline theta tACS during a post-encoding window of early memory consolidation, compared with three other conditions: frontal midline beta, posterior parietal theta, and sham. This benefit persisted up to a week following the intervention. Furthermore, in the free recall test conducted 2 h post-stimulation, the Frontal Theta group demonstrated a significant off-line gain in performance, which was not seen in the other three groups. While this off-line gain did not continue in subsequent assessments as it did in our NFB episodic study^9^, the difference might be attributable to the fact that in the current study, a 2-h break separated stimulation from the post-stimulation test. These findings are in consonance with previous studies^22^ pointing to the importance of frontal midline theta for mnemonic processes but extending them to show for the first time that theta power is important not only for encoding and retrieval but also for early stages of consolidation. In addition, results obtained in this paradigm indicate that the beneficial effects of theta upregulation, which we previously found in NFB studies^9,10,26^ cannot be attributed to strategic factors such as relaxation or manipulation of attentional focus. Rather, it indicates that theta oscillatory activity inherently benefits episodic memory consolidation processes.

In our prior work on episodic memory improvement via theta NFB^9,10^, the initial test to assess the effects of theta band upregulation on memory was conducted immediately after the NFB intervention. In the current study, the 2-h break between the stimulation and the fourth test was implemented as a washout period in order to separate the effects of stimulation on early consolidation from the lingering effects of such stimulation on retrieval. The fact that the Frontal Theta tACS group obtained improved memory performances, both initially and delayed, indicates that theta upregulation (via NFB or tACS) cannot be attributable to benefits to retrieval processes but rather to the consolidation of learning.

It should be noted that the differential benefit of frontal theta relative to parietal theta simulation sites should not be taken as indicating that there is a selective tACS effect on the specific brain region immediately below the frontal montage, as numerous studies have indicated that tACS has widespread effects throughout the brain and not only under the stimulating electrodes^34^. Rather, the frontal montage (inspired by findings regarding frontal midline theta relevance to memory processes) might have engendered more effective cortico-hippocampal oscillatory regulation than the parietal montage (which was inspired by findings regarding connectivity between the angular gyrus and the hippocampus in memory tasks).

As noted in Fig. 3, the montages and 1.5 mA intensity of stimulation employed in the current study yielded model estimates of relatively low electric field changes in the targeted areas: 0.12 mV/mm in the frontal montage and 0.28 mV/mm in the parietal montage. These are lower intensities than those usually required to engender tACS-induced neural entrainment (~0.3–0.4 mV/mm)^35^. However, even if not necessarily strong enough to induce entrainment, weak fluctuations in neural membrane potentials caused by lower-intensity tACS can bias spike timing^16,36^, in this case possibly contributing to the cortico-hippocampal communication at theta rhythms resulting from waking replay induced by prior learning. Some findings seem to indicate that larger modulations of spike timing at lower frequencies (in this case, theta) are possible at lower intensities than for higher frequencies^34^. It is also possible that the advantage of the frontal montage emerged since it is more focal, such that the actual electrical field changes were higher in the relevant area than the value yielded by the modeling.

Several limitations of this study should be noted. We were unable to measure the effects of scalp tACS on hippocampal activity. Therefore, we cannot know whether the observed memory effects of theta stimulation resulted from oscillatory modulation of post-encoding hippocampal or cortico-hippocampal activity or from some other processes. Future studies might provide such measurements, e.g., in pre-surgical epilepsy patients with electrodes implanted in the hippocampus. Another limitation is that the parietal montage we employed, which was designed to best modulate theta activity in the angular gyrus bilaterally, additionally stimulated other cortical and subcortical areas. Configurations with more localized stimulation specificity might yield different patterns of influences on memory consolidation. Another possibility that must be acknowledged is that the frontal theta tACS stimulation might have served to relax participants and quiet their minds, perhaps to a greater extent than parietal theta stimulation, and that having a relaxed mind following encoding is what served to benefit memory for the recently studied objects. This could come from either reducing interference from irrelevant thoughts and/or providing more opportunity for memory replay by not occupying the hippocampus with additional thoughts. Such an explanation could be an alternative to the idea that frontal theta tACS directly modulates the neural circuitry involved in memory consolidation.

While the current research could not explore the specific processes occurring on a neuronal level during and after transcranial stimulation, given prior findings of the importance of theta-band activity in both encoding and retrieval^37,38^, we speculate that theta entrainment might lead to greater waking replay of an encoding-related pattern of neural activity. This might have strengthened those memory representations, leading to better subsequent recall. Further research might explore this possibility, conceivably by conducting representational similarity analysis of hemodynamic activity patterns at encoding and retrieval in these experimental conditions. Additionally, it must be noted that prior research has indicated that theta tACS can engender complex patterns of changes in the scalp EEG, such as immediately subsequent increases in beta power^39^, and in the case of persistent application, alpha-beta phase locking values^40^. It may, therefore, be that theta stimulation engenders more effective consolidation through a range of cortico-hippocampal modulatory effects, not simply via theta power increases. As noted above, studies employing intra-cranial recordings might be able to determine which aspects of brain activity caused by theta stimulation are most crucial for the effective upregulation of consolidation processes.

## Methods

### Participants

Seventy-two volunteers (39 females; mean age 22.1 y, SD 2.5 y), all undergraduate students, took part in the study in return for payment and/or academic requirement credit. Exclusion criteria included any history of neurological or major psychological disorders, head injury, epilepsy (or any types of seizures), cardiac disease, pregnancy, or regular medication use. Additionally, persons with metal implants (including metal dental braces) or self-reported skin sensitivity in the head area were excluded. All participants reported having a minimum of 6 h of nocturnal sleep the night before and during the week of the experiment and no physiological sleep disruptions. Written informed consent was obtained from all participants for a protocol approved by the Institutional Review Board of Reichman University. Participants were assigned to one of the following groups, each comprising 18 participants: Theta Frontal group (11 F, 7 M, mean age 21.6 y, SD 2.5 y), Theta Parietal group (9 F, 9 M, mean age 23.1 y, SD 2.3 y), Beta Frontal group (10 F, 8 M, mean age 22.2 y, SD 2.3 y), or Sham group (9 F, 9 M, mean age 21.5 y, SD 2.7 y). In addition to the 72 participants mentioned above, six participants were excluded from the analyses since they performed more than 2.5 standard deviations above the mean of their groups in the pre-intervention baseline exam. Additionally, 13 participants were also excluded from the analysis since they were not able to complete the follow-up sessions of free recalls after 24 h and after 1 week (due to personal reasons, mostly related to the pandemic).

### Experimental design

**Fig. 2:**
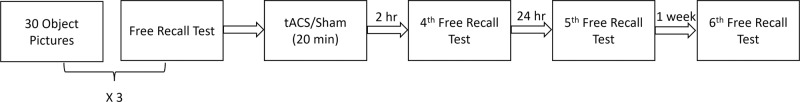
Experiment layout. Participants viewed 30 object pictures followed by a free recall test during three study-test cycles. They then engaged in tACS/Sham for a period of 20 min followed by a 2-h break. After the break, participants returned to the lab and took the fourth free recall test. The fifth free recall test took place 24 h later, and the sixth free recall test after 1 week; both longer-delay tests were conducted via a phone call.

The structure of the experiment is illustrated in Fig. 2. After assignment to one of the four experimental groups and before the initial learning, participants had the tACS headcap emplaced, to ensure that there was no time delay between the final pre-intervention study-test cycle and the start of the tACS protocol. After that preparation, participants were given task instructions and then viewed 30 object pictures (e.g., spoon, door, leaf, hammer, and bus) on a computer screen, each presented for 3 s. This was immediately followed by an oral free recall test, in which participants were given up to 5 min to recall as many of the viewed pictures as possible. Participants then viewed the 30 object pictures again in a different random order, followed by a second free recall test, which was followed by a third study-test cycle. Participants then engaged in tACS/sham for a period of 20 min. Before and after the stimulation participants’ pulse was measured to rule out states of psychological/physiological stress (i.e., within an acceptable target rate of 60–100 bpm). At the end of the stimulation procedure, participants were requested to fill in an adverse effects questionnaire to assess the tolerability of the stimulation^33^. A delay period of 2 h then ensued, during which time participants could engage in their usual campus activities (under conditions detailed below). Upon returning to the lab, they took the fourth recall test (without additional study beforehand). Twenty-four hours after the initial session, participants took the fifth recall text via phone call. This follow-up recall test was intended to determine the interaction of theta tACS and sleep for consolidation of episodic memory. One week after the initial session, participants took the sixth and final recall test (via phone, as before), intended to assess the stability of tACS effects. Experimenters assessing learning and memory were blind to the assignment of participants to stimulation conditions (theta/beta tACS or Sham), and participants were unaware of the goals of the experiment.

The 2-h break between stimulation and test was implemented to separate the effects of stimulation on early consolidation from the lingering effects of such stimulation on retrieval processes. We took several steps to prevent rehearsal during this and subsequent delay periods. Before participants were released for the break, they were told “Once you return to the lab, you will be given a short task related to your break”. After they returned from their break, they were told, “As mentioned before the break, you are now going to perform a short task related to your break. Before we start with the task, let us do a free recall of the items in the presentation”. The task was to report to the experimenter what they did during the break (1. “Attended a lecture\recitation” 2. “Studied but did not attend a lecture\recitation” 3. “Did not attend a lecture\recitation or study in any other setting”). We ensured that there was no significant difference between groups in the distribution of the type of activity in which they engaged during this break: *χ*^2^(9,72) = 1.26, *p* = 0.97. After the first day’s procedures, participants were told that for the second day of the experiment: “We will carry out the next part of the experiment tomorrow, at which time you will be given a short reading task”. After they completed the fifth recall test, we asked them to read a short paragraph (about a neutral subject: soil science) and told them that they would be tested on the paragraph in the final meeting, 1 week after the first session. This was intended to prevent differential conscious rehearsal of the object-free recall stimuli by focusing participants’ attention on another task that they believed would be the focus of their next assessment. After the sixth and final free recall test, we asked the participants what they remembered from the paragraph they read 6 days ago.

### tACS protocol

**Fig. 3:**
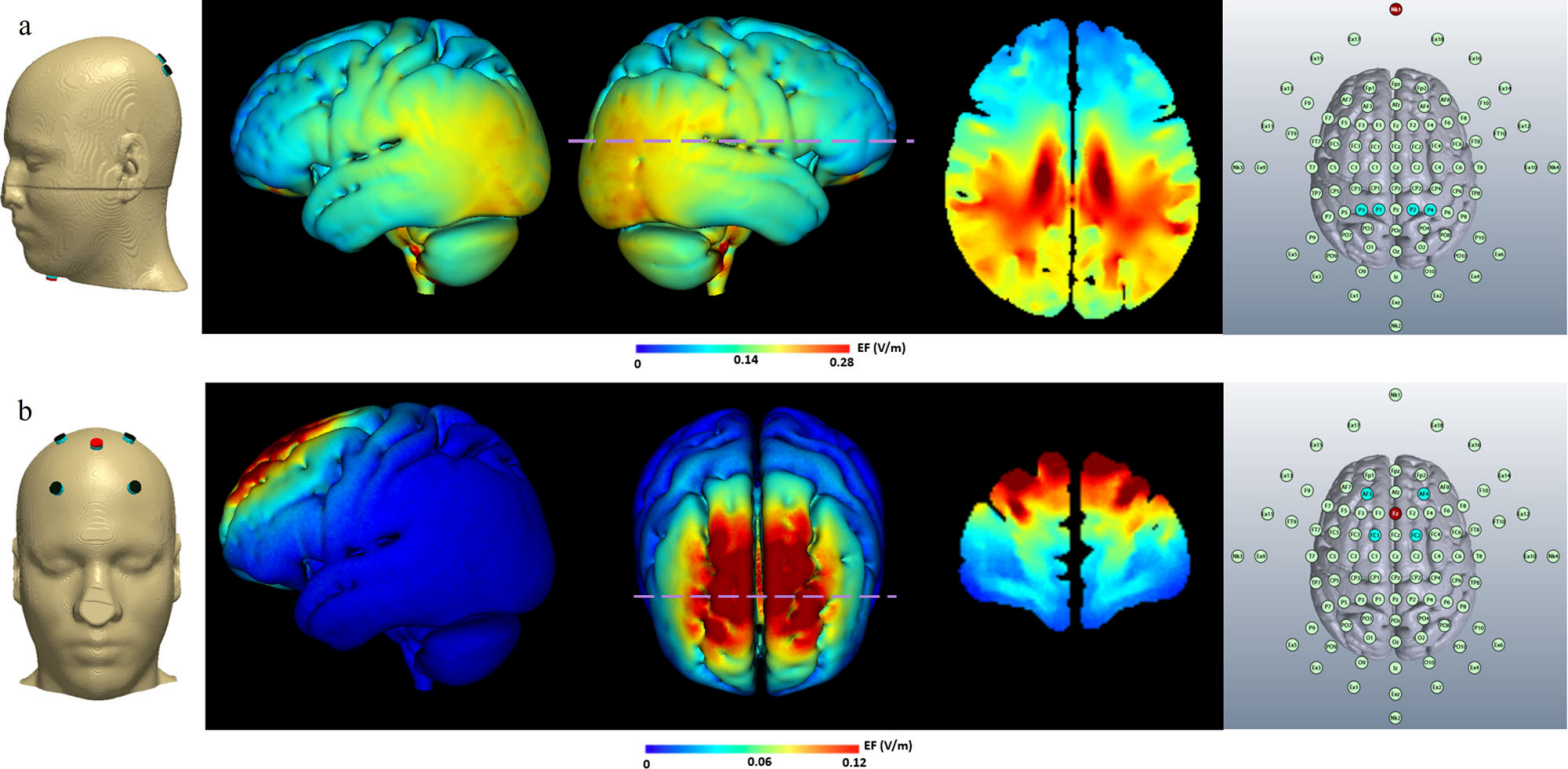
Current flow modeling during 1.5 mA HD-tACS using the HD-Target software (Soterix Medical, New York, NY). Electrodes’ locations and current-flow models and scales of the (**a**) parietal and (**b**) frontal montages are shown on 3D (left and center panels) and 2D (right panel) reconstructions.

A battery-driven, constant-current generator connected to a passive 4 × 1 HD-tDCS distributor (Soterix Medical Inc., New York, USA) was used to deliver alternating current to the scalp via five small circular electrodes, which have a better restraining effect on the electric field distribution of different phantom layers than larger rectangular electrodes^41^. We employed a ring montage, which can stimulate the target region more intensively than the classical bipolar electrode montage^42^. Stimulation was focused on different loci for the three active conditions of the experiment (Frontal Theta, Frontal Beta, and Parietal Theta). Electrode sites were selected using HD-Targets software (Soterix Medical Inc., New York, USA), which uses a finite-element model of a template adult brain to estimate the current distribution (Fig. 3). The algorithms implemented in that software are described in several reports^43–45^. For the Theta Parietal group, the angular gyrus (using an MNI 152 head model focus of ±37 [for bilateral coverage], −67, 30) was targeted using electrodes placed at positions P1–P4 of the International 10–20 system, with the return electrode placed on the chin. Recursive modeling using the Targets-HD software indicated that this montage optimally stimulated the angular gyrus bilaterally, although other brain regions were stimulated as well. For the Theta Frontal group, Fz was selected for the central electrode, based on the implication of midline frontal theta in memory processes^46^, specifically in episodic memory encoding and retrieval^22^. Furthermore, in our previous studies^9,10,26^ the Fz electrode was employed to provide real-time NFB, such that an Fz-focused montage would provide optimal comparisons of effects. The complementary ring electrodes for the Theta and Beta Frontal groups‘ montage were AF4, AF3, FC2, and FC1.

To reduce the contact impedance, a conductive gel (HD-GEL™, Soterix Medical Inc, New York, USA) was applied under the electrodes. The 4 × 1 Multichannel Stimulation Adapter Soterix device measures impedance in “quality units” (qu)^47^, which were kept under 1 qu for each electrode, which produced values of between 8 and 10 “contact quality” throughout the stimulation session. Stimulation time was set to 20 min, with a 30-s ramp-up and a 30-s ramp-down time for the three tACS groups (Frontal Theta group, Frontal Beta group, and Parietal Theta group). During sham stimulation, the current was ramped up to the pre-determined intensity level of 1.5 mA for 30 s, prior to being ramped down over the next 30 s to 0 mA, where it remained for the following 20 min. At the end of the sham session, the current was again ramped up to the pre-determined intensity level over 30 s. This procedure is commonly used to blind participants in TES studies^48,49^. The stimulation frequency for the Frontal Theta group and the Parietal Theta group was set at 3 Hz, which was found in prior studies to be the theta band frequency most strongly implicated in episodic memory^37,38^. For the Frontal Beta group, stimulation was set at 16 Hz^50^. The stimulation was delivered at an amplitude of 1.5 mA peak-to-peak. The stimulation demonstration, which was applied by using the “PRE-STIM TICKLE” feature of the Soterix constant-current generator just before the stimulation intervention, was delivered at an amplitude of 1.5 mA peak-to-peak for 30 s.

For the 20 min of tACS/Sham, participants were asked to stare at the blank computer screen in front of them and to keep their eyes open. An experimenter was always present in the room with the participants to monitor compliance but did not engage in conversation.

### Questionnaire regarding sensations related to transcranial electrical stimulation

After participants engaged in tACS/Sham for a period of 20 min, they completed a questionnaire regarding sensations related to electrical stimulation^33^. This questionnaire included questions about the level of itching, pain, burning sensation, warmth/heat, metallic/iron taste, fatigue/decreased alertness, etc., that the participants felt during the stimulation. The questionnaire also checked when the participants thought that the stimulation started, how long it lasted, how much it affected their general state and the location of the perceived sensation^33^. Importantly, this enabled us to assess whether participants knew if they were in sham or active groups.

### Statistical analysis

Statistical calculations were performed with SPSS 27 statistical software (IBM; Armonk, New York). All statistically significant analyses were set at *p* < 0.05. Visual inspections of q–q plots of residuals and estimates, as well as the Kolmogorov–Smirnov and Shapiro–Wilk tests, were used to examine the assumed normal distribution. tACS/Sham effects on memory performance were examined by repeated measures ANOVA. Effect sizes were calculated using Hedges’ *g*. For the reported sensations related to tACS, we used Univariate GLM analysis. The chi-square test was used to examine if participants were blind to the condition they were assigned, as well as possible differences between groups in the distribution of the type of activity in which they engaged during the 2 h break.

### Reporting summary

Further information on research design is available in the Nature Research Reporting Summary linked to this article.

### Supplementary information

Reporting summary

## Data Availability

This study’s datasets may be accessed at 10.6084/m9.figshare.24901584.
