# Supplementary material for: Frontal midline theta transcranial alternating current stimulation enhances early consolidation of episodic memory
Source: NPJ Sci Learn. 2024 Feb 16;9:8. doi: 10.1038/s41539-024-00222-0 (PMC10873319; doi:10.1038/s41539-024-00222-0)
Supplement: Supplementary file 1 — Reporting summary [file 41539_2024_222_MOESM1_ESM.pdf]

nature portfolio

Corresponding author(s): Daniel A. Levy

Last updated by author(s): 24-Dec-2023

Reporting Summary

Nature Portfolio wishes to improve the reproducibility of the work that we publish. This form provides structure for consistency and transparency in reporting. For further information on Nature Portfolio policies, see our Editorial Policies and the Editorial Policy Checklist.

Statistics

For all statistical analyses, confirm that the following items are present in the figure legend, table legend, main text, or Methods section.

n/a	Confirmed	
☐	☒	The exact sample size (*n*) for each experimental group/condition, given as a discrete number and unit of measurement
☐	☒	A statement on whether measurements were taken from distinct samples or whether the same sample was measured repeatedly
☐	☒	The statistical test(s) used AND whether they are one- or two-sided<br>*Only common tests should be described solely by name; describe more complex techniques in the Methods section.*
☒	☐	A description of all covariates tested
☐	☒	A description of any assumptions or corrections, such as tests of normality and adjustment for multiple comparisons
☐	☒	A full description of the statistical parameters including central tendency (e.g. means) or other basic estimates (e.g. regression coefficient) AND variation (e.g. standard deviation) or associated estimates of uncertainty (e.g. confidence intervals)
☐	☒	For null hypothesis testing, the test statistic (e.g. *F*, *t*, *r*) with confidence intervals, effect sizes, degrees of freedom and *P* value noted<br>*Give P values as exact values whenever suitable.*
☒	☐	For Bayesian analysis, information on the choice of priors and Markov chain Monte Carlo settings
☒	☐	For hierarchical and complex designs, identification of the appropriate level for tests and full reporting of outcomes
☐	☒	Estimates of effect sizes (e.g. Cohen's *d*, Pearson's *r*), indicating how they were calculated

Our web collection on statistics for biologists contains articles on many of the points above.

Software and code

Policy information about availability of computer code

Data collection	No software was used.
Data analysis	SPSS 27 statistical software (IBM; Armonk, New York).

For manuscripts utilizing custom algorithms or software that are central to the research but not yet described in published literature, software must be made available to editors and reviewers. We strongly encourage code deposition in a community repository (e.g. GitHub). See the Nature Portfolio guidelines for submitting code & software for further information.

Data

Policy information about availability of data

All manuscripts must include a data availability statement. This statement should provide the following information, where applicable:

- Accession codes, unique identifiers, or web links for publicly available datasets
- A description of any restrictions on data availability
- For clinical datasets or third party data, please ensure that the statement adheres to our policy

This study’s datasets may be accessed at the following link: https://doi.org/10.6084/m9.figshare.24901584.

Research involving human participants, their data, or biological material

Policy information about studies with human participants or human data. See also policy information about sex, gender (identity/presentation), and sexual orientation and race, ethnicity and racism.

Reporting on sex and gender	sex was not considered in the study design. Sex ratio was approximately even (39/72 participants F), and in each of the 4 experimental groups despite random allocation there was an approximately equivalent number of F and M: Theta Frontal group (11 F, 7 M, mean age 21.6 y, SD 2.5 y), Theta Parietal group (9 F, 9 M, mean age 23.1 y, SD 2.3 y), Beta Frontal group (10 F, 8 M, mean age 22.2 y, SD 2.3 y), or Sham group (9 F, 9 M, mean age 21.5 y, SD 2.7 y).
Reporting on race, ethnicity, or other socially relevant groupings	No socially constructed variables were employed in this study. All participants in this between-participants design study came from the same recruitment base and were allocated randomly to experimental groups.
Population characteristics	See above.
Recruitment	Participants were recruited in a university experiment participation website.
Ethics oversight	Institutional Review Board of Reichman University

Note that full information on the approval of the study protocol must also be provided in the manuscript.

Field-specific reporting

Please select the one below that is the best fit for your research. If you are not sure, read the appropriate sections before making your selection.

☐ Life sciences ☒ Behavioural & social sciences ☐ Ecological, evolutionary & environmental sciences

For a reference copy of the document with all sections, see nature.com/documents/nr-reporting-summary-flat.pdf

Behavioural & social sciences study design

All studies must disclose on these points even when the disclosure is negative.

Study description	Quantitative
Research sample	undergraduate students
Sampling strategy	Sample size was identical to the sample size of our prior research in this topic.
Data collection	Free recall memory performance was recorded with paper and pen by experimenters. Responses were voice recorded, and scoring was confirmed in comparison with those recordings. Experimenters assessing learning and memory were blind to the assignment of participants to stimulation condition, and participants were unaware of the goals of the experiment.
Timing	Data was collected continuously during 2021.
Data exclusions	Six participants were excluded from the analyses since they performed more than 2.5 standard deviations above the mean of their groups in the pre-intervention baseline exam. Additionally, 13 participants were also excluded from the analysis since they were not able to complete the follow-up sessions of free recalls after 24 hours and after 1 week (due to personal reasons, mostly related to the pandemic).
Non-participation	None
Randomization	Random allocation in order of recruitment.

Reporting for specific materials, systems and methods

We require information from authors about some types of materials, experimental systems and methods used in many studies. Here, indicate whether each material, system or method listed is relevant to your study. If you are not sure if a list item applies to your research, read the appropriate section before selecting a response.

Materials & experimental systems

n/a	Involved in the study
☒	☐ Antibodies
☒	☐ Eukaryotic cell lines
☒	☐ Palaeontology and archaeology
☒	☐ Animals and other organisms
☒	☐ Clinical data
☒	☐ Dual use research of concern
☒	☐ Plants

Methods

n/a	Involved in the study
☒	☐ ChIP-seq
☒	☐ Flow cytometry
☒	☐ MRI-based neuroimaging

Plants

Seed stocks	*Report on the source of all seed stocks or other plant material used. If applicable, state the seed stock centre and catalogue number. If plant specimens were collected from the field, describe the collection location, date and sampling procedures.*
Novel plant genotypes	*Describe the methods by which all novel plant genotypes were produced. This includes those generated by transgenic approaches, gene editing, chemical/radiation-based mutagenesis and hybridization. For transgenic lines, describe the transformation method, the number of independent lines analyzed and the generation upon which experiments were performed. For gene-edited lines, describe the editor used, the endogenous sequence targeted for editing, the targeting guide RNA sequence (if applicable) and how the editor was applied.*
Authentication	*Describe any authentication procedures for each seed stock used or novel genotype generated. Describe any experiments used to assess the effect of a mutation and, where applicable, how potential secondary effects (e.g. second site T-DNA insertions, mosiacism, off-target gene editing) were examined.*
